# Rapid measurement of thoracolumbar kyphosis with the integrated inclinometer of a smartphone: a validity and reliability study

**DOI:** 10.1038/s41598-022-12690-8

**Published:** 2022-05-24

**Authors:** Tianji Huang, Zenghui Zhao, Lin Wang, Chao Zhang, Runhan Zhao, Chuang Xiong, Weiyang Zhong, Xiaoji Luo

**Affiliations:** Department of Orthopedic Surgery, The First Affiliated Hospital of Chongqing Medical University, Chongqing, 400016 People’s Republic of China

**Keywords:** Medical imaging, Trauma, Orthopaedics, Musculoskeletal system, Pain, Musculoskeletal system

## Abstract

The objective of this study was to evaluate the accuracy, reliability, and time requirements of two methods for measuring thoracolumbar kyphosis: a conventional method using a picture archiving and communication system (PACS) and this new method using the integrated inclinometer of a smartphone. The thoracolumbar kyphotic angles of one hundred consecutive patients were measured by a PACS and this novel smartphone method. The measured angles were analysed by multiple statistical methods, and the two measurement tools were compared in terms of accuracy, reliability, and time requirements. The mean result of thoracolumbar kyphosis measured by the PACS was 21.43 ± 12.96°, and the mean value measured by the smartphone was 21.03 ± 13.01°. A Bland–Altman plot for these two methods showed a mean difference of 0.4°, with the limits of agreement being -2.4° and 3.2°. One-way ANOVA showed no significant difference (F = 0.080, *P* = 0.999) among measurements by different observers and different methods. The intraclass correlation coefficients (ICCs) of the mean values of four repeated measurements of thoracolumbar kyphosis between these two methods were 0.997 (0.995–0.998), revealing that the two methods were highly correlated. The ICC results showed that the concordance between these two methods was very good for all measurements of thoracolumbar kyphosis, and the inter- and intra-observer reliability of the novel smartphone method were very good. The PACS method (36.95 ± 0.98 s) took significantly longer than the smartphone method (17.68 ± 0.97 s) when compared by an independent-samples t test (*P* = 0.000). This new method using the integrated inclinometer of a smartphone has satisfactory validity and reliability compared to the PACS method. Additionally, the new method took significantly less time than the PACS method. Measuring with a smartphone is more convenient than using a PACS, which is always rooted in a full-sized computer. In summary, this new method using the integrated inclinometer of a smartphone is rapid, convenient, accurate and reliable when measuring thoracolumbar kyphosis in osteoporotic vertebral compression fracture (OVCF) patients.

## Introduction

Osteoporotic vertebral compression fractures (OVCFs) may result in severe thoracolumbar kyphosis, which presents several challenges to spine surgeons^1^. The thoracolumbar kyphosis of patients with OVCFs at the thoracolumbar junction is markedly greater than that of healthy volunteers and can decrease significantly after percutaneous kyphoplasty^2^. Accurate measurement of thoracolumbar kyphosis in patients with thoracolumbar OVCFs is helpful for selecting treatment options and predicting prognosis. The traditional measuring method, in which a marking pen and protractor are used, is inconvenient and time-consuming, and could be more troublesome and film-stained when using included angle of vertical lines of endplates in small kyphotic angle patients^3^. The current gold standard relies on picture archiving and communication systems (PACSs), which are normally rooted in hospital computer systems and lack portability as well as convenient accessibility by a large user base^4^. Some iPhone apps have been designed and used to measure kyphotic angles in lateral X-ray films; however, these apps have some unavoidable disadvantages, including fees and lack of software stability^5,6^. The inclinometer, which is a built-in function in almost every smartphone, can be used to measure the inclination of the smartphone during photography or other circumstances. We introduced a new method using the integrated inclinometer of a smartphone to measure thoracolumbar kyphosis and analysed the accuracy and reliability of this new method.

## Materials and methods

All methods were carried out in accordance with relevant guidelines and regulations. This study was approved by the ethics committee of The First Affiliated Hospital of Chongqing Medical University, which waived the requirement for informed consent.

### Patient selection

One hundred consecutive patients admitted to our hospital and diagnosed with OVCFs were retrospectively reviewed. The inclusion criteria were legible lateral plain X-ray films and one or more acute or chronic osteoporotic vertebral compression fractures at the thoracolumbar junction (T10-L2). The exclusion criteria were spinal tumours, spinal tuberculosis, and pyogenic spinal infections.

### Measurement methods

**Figure 1:**
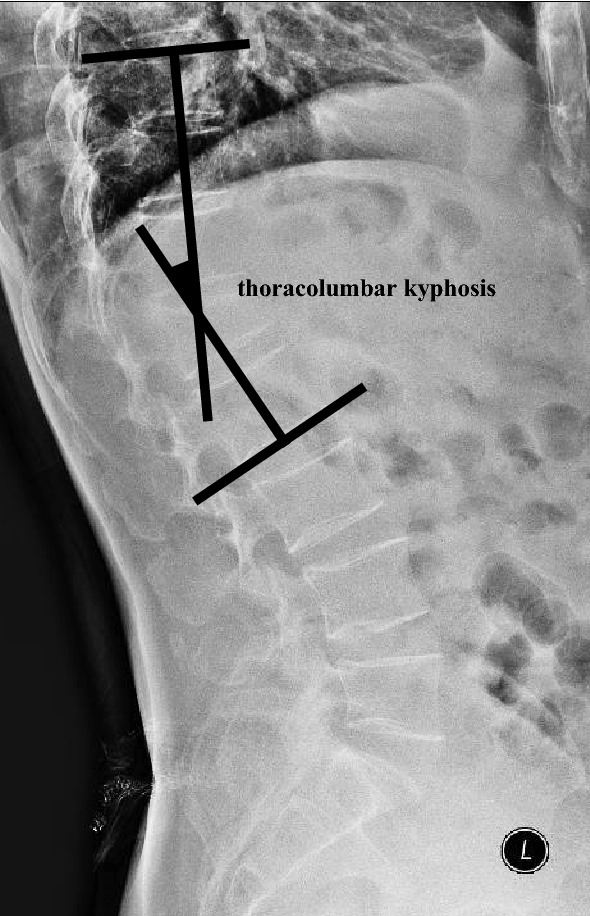
Measurement of thoracolumbar kyphosis. After the vertebral bodies of T10 and L2 were identified, a line was drawn overlapping the upper endplate of T10, and another line was drawn overlapping the lower endplate of L2; the angle between the two lines represented the degree of thoracolumbar kyphosis.

Thoracolumbar kyphosis was defined as the included angle between the upper endplate of T10 and the lower endplate of L2 (or the included angle between vertical lines normal to the two aforementioned endplates)^2,5^ (Fig. 1).

Thoracolumbar kyphosis measurement with a PACS was implemented by using the PACS’s integrated system to mark lines manually and read them automatically.

**Figure 2:**
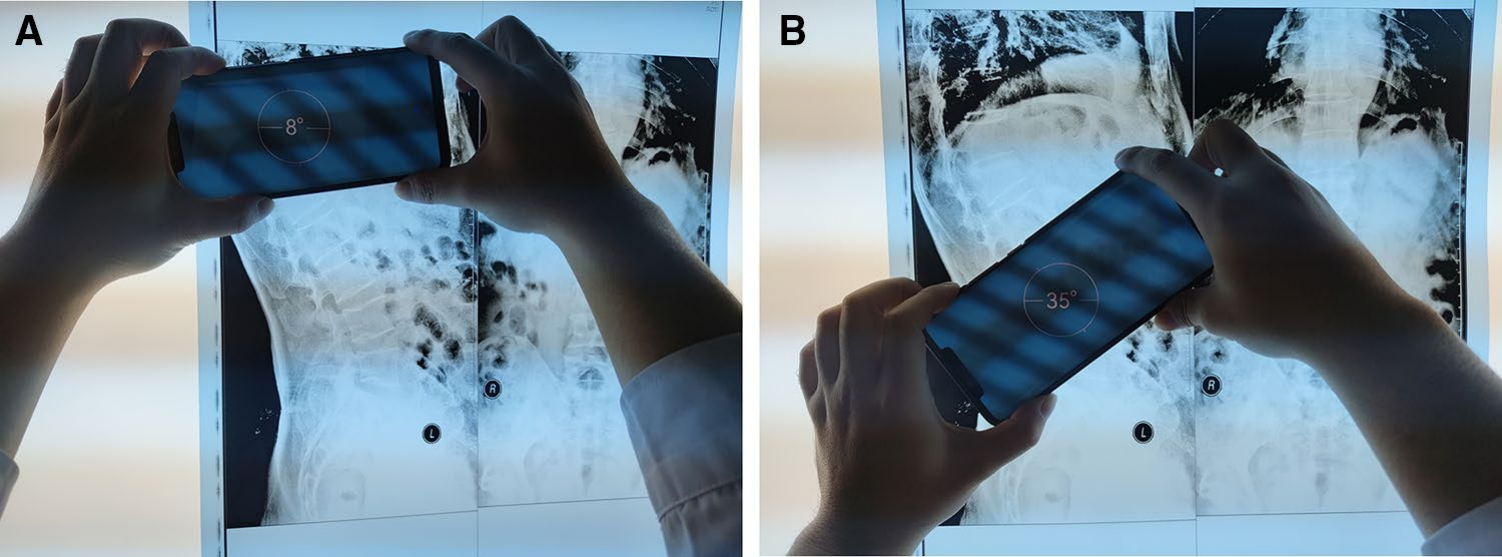
The integrated inclinometer in a smartphone was used to measure thoracolumbar kyphosis on lateral plain X-ray films. (**A**) First, the vertebral body of T10 was confirmed, a certain edge of the smartphone was aligned to the upper endplate of T10, and the angle shown on the smartphone screen was recorded. (**B**) Second, the vertebral body of L2 was confirmed, the same edge of the smartphone was aligned to the lower endplate of L2, and the angle shown on the smartphone screen was recorded.

The smartphone used in the study is a HUAWEI Mate 20 Pro. The resolutions of the three Leica rear cameras are 40 million, 20 million and 8 million pixels. The inclinometer build into this smartphone was used to measure thoracolumbar kyphosis on lateral plain X-ray film. The precision of the built-in inclinometer sensor is one degree. The X-ray film was placed on the film viewer, which was located on the wall; hence, the X-ray film was assumed to be plumb (Fig. 2). First, the vertebral body of T10 was confirmed, the certain edge of the smartphone was aligned to the upper endplate of T10, and the angle shown on the smartphone screen was recorded (Fig. 2A). Second, the vertebral body of L2 was confirmed, the same edge of the smartphone was aligned to the lower endplate of L2, and the angle shown on the smartphone screen was recorded (Fig. 2B). Third, we calculated and recorded the difference between these two angles, which represented thoracolumbar kyphosis. Because the smartphone is not transparent, errors may be produced when aligning the edge of the smartphone to the endplate line; observers should try to avoid this potential error.

Without knowledge of the patients’ information, two observers (observer A is an attending spine surgeon, and observer B is an orthopaedic resident undergoing training in general orthopaedics) who had undergone sufficient training and practice with this new method independently evaluated the X-ray films. Each angle obtained by the PACS and smartphone was measured twice over 2 weeks. The order of the four measurements was as follows: first trial with the PACS (first week), first trial with the smartphone (third week), second trial with the PACS (fifth week), and second trial with the smartphone (seventh week). To decrease possible recall, the order of the radiographs was reorganized randomly using the RAND function in Excel software.

A stopwatch was used to record the time taken for each measurement. To simulate the actual measurement process used in the clinic, the measurement time of the PACS began when the radiograph number was entered in the PACS and ended when the angle was recorded; the measurement time of the smartphone began at the activation of the integrated inclinometer function and ended when the angle was recorded.

Excel 2016 was used to record a total of 800 (one hundred thoracolumbar junction × two methods × two times × two observers) thoracolumbar kyphotic angles.

### Statistical analysis

Statistical analysis was performed in a blinded manner. All the data were analysed by SPSS 21.0. The Kolmogorov–Smirnov test was used to check whether the values were normally distributed. Bland–Altman analysis was performed in MedCalc software to evaluate the agreement between the mean values by these two methods. One-way ANOVA was used to evaluate the differences among all measurements by different observers and different methods. The reliability of the two measurement methods was analysed by two-way random intraclass correlation coefficients (ICCs). ICC values divide reliability into five levels: poor (0.00–0.20), fair (0.21–0.40), moderate (0.41–0.60), substantial or good (0.61–0.80) and very good (0.81–1.00). The time needed for different measurement methods was also compared by an independent-samples t test. The significance threshold was set at 0.05.

## Results

This study reviewed 100 lateral radiographs from osteoporotic vertebral compression fracture patients (12 males and 88 females). The average age of all the patients was 71.8 ± 9.00 years.

**Figure 3:**
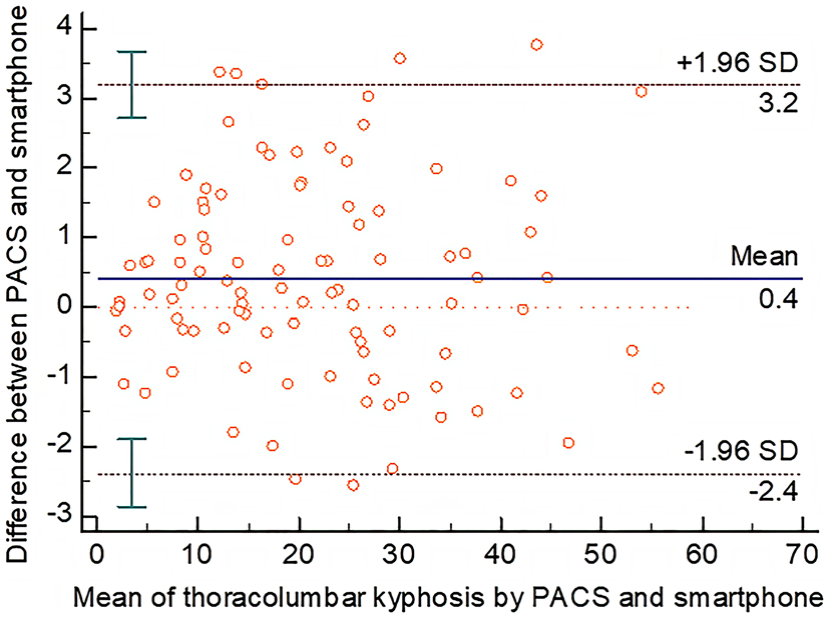
Bland–Altman plot. The solid line in the centre represents the mean difference, and the two outer dotted lines represent the limits of agreement (95% confidence interval).

All measurements of thoracolumbar kyphosis were normally distributed, as confirmed by the Kolmogorov–Smirnov test. The mean result of thoracolumbar kyphosis measured by the PACS was 21.43 ± 12.96°, and the mean result of thoracolumbar kyphosis measured by the smartphone was 21.03 ± 13.01°. Figure 3 shows the Bland–Altman plot with the mean difference between these two methods (0.4°) and the limits of agreement (− 2.4° and 3.2°). Most of the data were within the limits of agreement, indicating that the agreement of these two methods is good. One-way ANOVA showed no significant differences (F = 0.080, *P* = 0.999) among the measurements by different observers and different methods (Table 1). The ICC of the mean values of the four measurements of thoracolumbar kyphosis between these two methods was 0.997 (0.995–0.998), revealing that these two methods were highly correlated. Table 2 shows that the concordance between these two methods was very good for every measurement of thoracolumbar kyphosis. Table 3 shows that the inter- and intra-observer reliability of thoracolumbar kyphosis measurement with this novel smartphone method were very good. The time needed to use the PACS (36.95 ± 0.98 s) was significantly longer than the time needed to make the same measurement with a smartphone (17.68 ± 0.97 s), as compared by an independent-samples t test (*P* = 0.000).

**Table 1:** Mean ± SD values of all measurements and their comparison.

	Thoracolumbar kyphosis (°)
PACS A t1	21.33 ± 13.47
PACS A t2	20.99 ± 12.93
PACS B t1	21.58 ± 13.42
PACS B t2	21.83 ± 12.89
Smartphone A t1	20.70 ± 13.32
Smartphone A t2	21.05 ± 13.12
Smartphone B t1	20.96 ± 12.82
Smartphone B t2	21.39 ± 13.16
Significance (*P* value)	0.999

*P* values indicated the significance between the values in the same column.

PACS A t2: the results of the second time of observer A using PACS, and so on.

Smartphone B t1: the results of the first time of observer B using smartphone, and so on.

**Table 2:** The reliability of results using PACS and smartphone of every measurement.

	ICC (95% CI)	*P* value
**Observer A t1 PACS versus smartphone**		
Thoracolumbar kyphosis	0.965 (0.948–0.976)	0.000
**Observer A t2 PACS versus smartphone**		
Thoracolumbar kyphosis	0.968 (0.952–0.978)	0.000
**Observer B t1 PACS versus smartphone**		
Thoracolumbar kyphosis	0.958 (0.938 -0.971)	0.000
**Observer B t2 PACS versus smartphone**		
Thoracolumbar kyphosis	0.963 (0.946–0.975)	0.000

**Table 3:** Inter- and intraobserver reliability of observer A’s versus observer B’s measurements using smartphone.

	ICC (95% CI)	*P* value
**Observer A t1 versus observer B t1**		
Thoracolumbar kyphosis	0.977 (0.966–0.985)	0.000
**Observer A t2 versus observer B t2**		
Thoracolumbar kyphosis	0.975 (0.962–0.983)	0.000
**Observer A t1 versus observer A t2**		
Thoracolumbar kyphosis	0.984 (0.976–0.989)	0.000
**Observer B t1 versus observer B t2**		
Thoracolumbar kyphosis	0.982 (0.974–0.988)	0.000

Observer A t2: the results of the second time of observer A, and so on.

Observer B t1: the results of the first time of observer B, and so on.

## Discussion

The traditional manual method and the PACS method have their own advantages and disadvantages in measuring spinal parameters^7^. Electronic devices, especially smartphones, have been used in evaluating different situations in orthopaedic patients. Some apps have been used to measure the range of motion of the shoulder^8^, elbow^9^, wrist^10^ and knee^11^. The motion of the cervical^12^, thoracic^13^ and lumbar spine^14^ has also been evaluated by various types of iPhone apps.

Some iPhone apps have been introduced to measure Cobb angles on lateral radiographs in different clinical conditions. Jacquot et al. developed an iPhone app called Cobbmeter and proved the excellent reliability of this new app, which was found to be non-inferior to the traditional manual method for measuring Cobb angles on lateral X-ray films^5^. Lee et al. compared the “Sagittalmeter Pro” app to the PACS in measuring spinopelvic sagittal parameters, and the results showed that this app had good intra- and inter-rater reliability, indicating that the app is useful and time saving when planning spinal surgery^6^. Shahri et al. validated the Goniometer-Pro app as a highly accurate and reliable method for measuring thoracic curvature values^15^.

However, smartphone apps have their own disadvantages when measuring these kyphotic parameters of the spine. The lack of updating and routine maintenance make these apps incompatible with the latest smartphone. Extra fees and potential bugs make them not as friendly as the built-in function of smartphones^4^. Most of these apps are based on the Apple iPhone platform, thus excluding users of Android and other smartphone systems, which are common in developing countries.

Therefore, we presented a new method using the integrated inclinometer, which is found in almost every smartphone system, for measuring thoracolumbar kyphosis in spinal lateral radiography. The accuracy and reliability of this new method were tested in the present study. The results indicated that the validity and reliability of this new method are excellent and that the time requirement of this novel method is notably less than that of the PACS method.

Notably, the Bland–Altman plot showed that the mean difference between these two methods was less than 11°, which is a widely acceptable error in the measurement of kyphotic angles in the spine^16,17^. Possible errors from this novel method may come from the confirmation of the endplate due to the poor quality of radiography, which can also be seen in the manual method and PACS method. Because the inclinometer function is built into the smartphone, this novel method avoids the aforementioned problems with downloaded apps. It does not need to be updated or maintained, always responds quickly without bugs, and is included with the phone at no additional charge^18^. The purpose and the spirit of the measurement have not been changed even though the method has changed; a new tool has been developed, but the general principles of the kyphotic measurement process remain^19^. This novel method is not intended to replace the traditional manual method and PACS method but to provide a new possibility for rapid and convenient measurement.

In the present study, we evaluated the use of a built-in smartphone inclinometer for assessing thoracolumbar kyphosis only in OVCF patients due to the ease of obtaining data from these patients. Because of the similar principles and steps, this method may be useful in other spinal parameter measurements. Even though the results of this study are encouraging, more research is needed to confirm the universal feasibility in different clinical conditions. This study assessed only the scenario of measurement on X-ray films with this integrated inclinometer; its accuracy and reliability for measuring radiographs on a computer screen remain unclear. It can be reasonably speculated that if the computer screen is calibrated to be plumb, this method should also be suitable for the measurement of radiographs on the computer screen. This usefulness of this novel method in such a clinical situation may need further evaluation.

Additionally, some points deserve special attention during the measurement process. The X-ray films need to be placed plumb because the principle of this novel method is based on an inclinometer. Sufficient practice and proficiency are associated with measurement accuracy and time efficiency.

## Conclusion

The validity and reliability of this new method using an integrated smartphone inclinometer are satisfactory compared to those of the PACS method. The time requirement of this new method is significantly shorter than that of the PACS method. Measuring with smartphones is more convenient than using a PACS, which is always rooted with a full-sized computer. In summary, this new method using the integrated inclinometer of a smartphone is rapid, convenient, accurate and reliable for measuring thoracolumbar kyphosis in OVCF patients.

## Data Availability

The datasets generated and/or analysed during the current study are not publicly available due to further research but are available from the corresponding author on reasonable request.

## Abbreviations

PACS: Picture archiving and communication system
OVCFs: Osteoporotic vertebral compression fractures
ICCs: Intraclass correlation coefficients
